# Long Non-coding RNA CASC2 Enhances the Antitumor Activity of Cisplatin Through Suppressing the Akt Pathway by Inhibition of miR-181a in Esophageal Squamous Cell Carcinoma Cells

**DOI:** 10.3389/fonc.2019.00350

**Published:** 2019-05-07

**Authors:** Dengyan Zhu, Yang Yu, Yu Qi, Kai Wu, Donglei Liu, Yang Yang, Chunyang Zhang, Song Zhao

**Affiliations:** ^1^Department of Thoracic Surgery, The First Affiliated Hospital of Zhengzhou University, Zhengzhous, China; ^2^Department of Anesthesiology, The First Affiliated Hospital of Zhengzhou University, Zhengzhou, China

**Keywords:** esophageal squamous cell carcinoma, CASC2, miR-181a, cisplatin, Akt pathway

## Abstract

**Background:** Long non-coding RNA CASC2 (lncRNA CASC2) has been found to be down-regulated in esophageal squamous cell carcinoma (ESCC). However, the effect of CASC2 on cisplatin-treated ESCC was unclear. The present study aimed to evaluate the role of CASC2 in cisplatin-treated ESCC cells.

**Methods:** The expression levels of CASC2 and miR-181a were detected by qRT-PCR. Cell viability was measured by MTT assay. The cytotoxicity effect was detected by lactate dehydrogenase (LDH) release assay. Cell apoptosis was tested by flow cytometry. The protein levels of protein kinase B (Akt) and p-Akt were detected by western blotting.

**Results:** The results showed that CASC2 was low-expressed in ESCC cell lines. Overexpression of CASC2 enhanced the inhibitory effect of cisplatin on cell viability and promoted cisplatin-induced LDH release and apoptosis. We also found that miR-181a expression levels were increased in ESCC cell lines. MiR-181a inhibitor enhanced the antitumor activity of cisplatin, which was similar with the effect of CASC2. CASC2 directly interacted with miR-181a and inhibited the miR-181a expression. MiR-181a reversed the effects of CASC2 on antitumor activity of cisplatin. In addition, we also found that CASC2 suppressed the Akt pathway by inhibiting miR-181a.

**Conclusions:** CASC2 promoted the antitumor activity of cisplatin through inhibiting Akt pathway via negatively regulating miR-181a in ESCC cells. The results provide a new insight for ESCC therapy.

## Introduction

The incidence of esophageal cancer ranks the eighth most common cancer worldwide and the mortality rate ranks the sixth (1). There are two main sub-types of the esophageal cancer, esophageal squamous-cell carcinoma (ESCC) and esophageal adenocarcinoma (EAC). ESCC is the major histologic subtype of esophageal cancer (2). The overall survival for ESCC patients is comparatively short, and the 5-year survival rate of ESCC is about 14% (2). Recently, molecular-targeted agents have been studied for the treatment of many diseases. The studies on ESCC are imperative.

Long non-coding RNA (lncRNA) is a group of RNAs that are defined as transcripts longer than 200 nucleotides but not translate into proteins. LncRNAs have been discovered to play important roles in regulating the developmental, physiological, and pathological processes in humans (3). Particularly, lncRNAs were found to be involved in the development and metastasis of various cancers, such as gastric cancer (4), ovarian cancer (5), and ESCC (6). LncRNA CASC2 has been reported to possess antitumor effect in many cancers, including hepatocellular carcinoma (7), gastric cancer (8), and cervical cancer (9). It has been reported that CASC2 is down-regulated in EC tissues and EC cell lines (10). However, the role of CASC2 in regulating cisplatin-treated ESCC is unclear.

MicroRNAs (miRNAs) are a group of small non-coding RNAs that participate in a series of biological events (11). MiRNAs function through regulating its target mRNAs (12). Since miRNAs play crucial roles in caner development, they have the potential to be used as medical targets for the treatment of many cancers (13, 14). It has been reported that lncRNAs have to interact with DNA, protein, mRNAs, and miRNAs to achieve their functions (15). To fully investigate the role of lncRNAs in ESCC, its target gene should also be understood.

The role of CASC2 and its target miRNA in cisplatin-treated ESCC cells was investigated in the present study. The results suggested that CASC2 was low-expressed and its target miRNA (miR-181a) was over-expressed in ESCC cell lines. CASC2 enhanced the antitumor effect of cisplatin via suppressing the protein kinase B (Akt) pathway.

## Materials and Methods

### Cell Culture

Human normal esophageal epithelial cell line Het-1A and human ESCC cell lines Eca109, KYSE140, KYSE150, TE-1, and EC9706 were obtained from the Cell Bank of Type Culture Collection of the Chinese Academy of Sciences (Shanghai, China). Cells were cultured in RPMI-1640 medium (Invitrogen, Carlsbad, CA, USA) supplemented with 10% fetal bovine serum (Gibco, USA) at 37°C with 5% v/v CO_2_.

### Transfection

Cells were cultured in 24-well plates and the transfection was performed using Lipofectamine 2,000 reagent (Invitrogen) according to the manufacturer's protocol. Vector containing CASC2 (pcDNA3.1-CASC2), vector alone (pcDNA3.1), an antisense inhibitor against miR-181a (miR-181a inhibitor), inhibitor control, miR-181a mimics, scramble mimic control (mimic control), a small interfering RNA (siRNA) against CASC2 (si-CASC2), and siRNA control were synthesized by GenePharma (Shanghai, China).

### qRT-PCR

Total RNA was isolated from cells using Trizol reagent (Invitrogen). One microgram of total RNA was used to synthesize cDNA using First Strand cDNA Synthesis Kit (Takara, Dalian, China). The SYBR Premix Ex Taq (Takara) was used for the qRT-PCR according to the manufacturer's instruction. The primers used are as follows: CASC2, forward 5′- GCAC ATTG GACG GTGT TTCC-3′, reverse 5′-CCCA GTCC TTCA CAGG TCAC-3′; U6, forward 5′-CTCG CTTC GGCA GCAC A-3′, reverse 5′- AACG CTTC ACGA ATTT GCGT-3′; GAPDH, forward 5′-GTCA ACGG ATTT GGTC TGTA TT-3′, reverse 5′-AGTC TTCT GGGT GGCA GTGA T-3′. The reaction conditions were as follows: denatured at 95°C for 5 min; followed by 35 cycles at 95°C for 20 s, annealing at 55°C for 30 s, extension at 72°C for 20 s. The expression of mRNA and miRNA was normalized to GAPDH or U6 expression levels, respectively. The expression fold changes were calculated with the 2^−ΔΔCt^ method.

### MTT Assay

The MTT assay was used to evaluate cell viability of TE-1 and EC9706. Briefly, transfected cells were plated into 96-well plates and incubated with cisplatin for 48 h. After incubation, MTT solution was added and incubated for 4 h. The formed formazan was dissolved with DMSO solution, and the absorbance was measured at 490 nm with a microplate reader (Bio-Rad Laboratories, CA, USA).

### Lactate Dehydrogenase (LDH) Release Assay

After different treatments, the cellular release of LDH was measured. Briefly, the supernatants from TE-1 and EC9706 cells were collected and the LDH activity was detected using a commercial kit (Nanjing Jiancheng Bioengineering Institute, Nanjing, China).

### Flow Cytometry

Flow cytometry was performed to detect cell apoptosis. After different treatments, cells were harvested and washed for three times. Then cells were incubated with Annexin V-FITC and propidium iodide in the dark for 15 min at room temperature. Cells were washed for three times and analyzed using a flow cytometer (FACS Calibur, BD Biosciences, Franklin Lakes, NJ, USA).

### Western Blotting

Cells were lysed using protein extraction reagent RIPA (Beyotime Biotechnology, Shanghai, China). The protein concentration was measured using a protein assay kit (Bio-Rad). The proteins (50 μg) were separated by 12% SDS-PAGE and transferred to nitrocellulose membranes. Subsequently, the membranes were blocked with 5% skim milk powder in Tris buffered saline for 2 h at room temperature. The membrane was incubated with specific primary antibodies against Akt (Abcam, Cambridge, MA, USA), p-Akt (S473) (Abcam), phosphorylated histone H2A.X (p-H2A.X; Ser139) (Cell Signaling Technology, Beverly, MA, USA), and β-actin (Abcam) overnight at 4°C. Then the membrane was incubated with a peroxidase-conjugated secondary antibody (Abcam) at room temperature for 2 h. The protein bands were visualized using the enhanced chemiluminescence (ECL) reagent (Bio-Rad). The band intensities were quantified using scanning densitometry by Quantity One Software (Bio-Rad). β-actin was used as an internal reference. The results are expressed as the ratio of the sample band intensity value to β-actin band intensity value, and the control data were set as 1.

### Luciferase Reporter Assay

TE-1 cells were seeded in a 24-well plate and cultured overnight. Cells were co-transfected with pmirGLO-CASC2-3′UTR-WT (wild type) or pmirGLO-CASC2-3′UTR-MUT (mutant type) and miR-181a mimics or mimic control according to the manufacturer's instructions. After 48 h, the luciferase activity was detected using the Dual-Luciferase Reporter Assay System (Promega, Madison, WI USA).

### Statistical Analysis

All experiments were performed at least three times. All results are presented as mean ± SD. The data were analyzed using SPSS version 22.0 software (IBM, Chicago, IL, USA). The one-way ANOVA was used to evaluate the differences among groups. *p* < 0.05 were considered statistically significant.

## Results

### LncRNA CASC2 Was Down-Regulated and CASC2 Overexpression Induced DNA Damage in ESCC Cells

**Figure 1:**
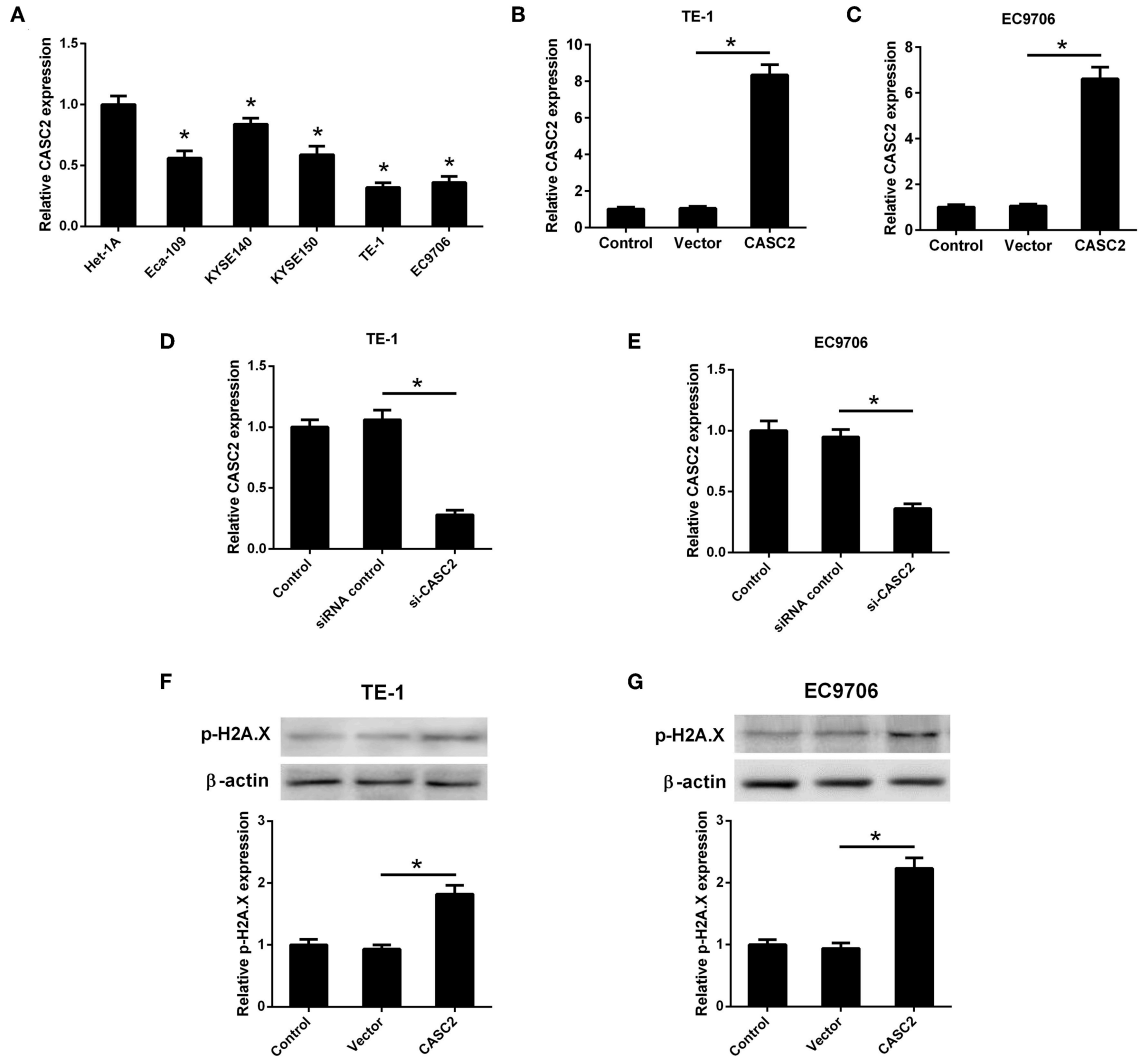
LncRNA CASC2 was down-regulated in ESCC cells. **(A)** The expression of CASC2 in normal esophageal epithelial cell line (Het-1A) and human ESCC cell lines (Eca109, KYSE140, KYSE150, TE-1, and EC9706) was detected by qRT-PCR. ^*^p < 0.05 vs. Het-1A cells, *n* = 3. **(B,C)** The expression of CASC2 in TE-1 and EC9706 cells transfected with pcDNA3.1-CASC2 (CASC2) or empty vector pcDNA3.1 (Vector) for 48 h. ^*^p < 0.05, *n* = 3. **(D,E)** The expression of CASC2 in TE-1 and EC9706 cells transfected with si-CASC2 or siRNA control for 48 h. ^*^p < 0.05, *n* = 3. **(F,G)** The levels p-H2A.X was determined using western blot analysis in TE-1 and EC9706 cells 48 after transfection with CASC2 or Vector. ^*^p < 0.05, *n* = 3.

The expression levels of CASC2 in Het-1A, Eca109, KYSE140, KYSE150, TE-1, and EC9706 were detected by qRT-PCR. The results in Figure 1A showed that CASC2 was low-expressed in human ESCC cell lines compared to normal esophageal epithelial cell line. Among the five ESCC cell lines, TE-1 and EC9706 cells exhibited lower expression levels of CASC2. Thus, TE-1 and EC9706 cells were selected for the further experiments. To evaluate the role of CASC2 in TE-1 and EC9706 cells, the CASC2 overexpression vector (pcDNA3.1-CASC2), empty vector (pcDNA3.1), siRNA targeting CASC2 (si-CASC2), and siRNA control were transfected into TE-1 and EC9706 cells. The expression levels of CASC2 in cells transfected with pcDNA3.1-CASC2 were significantly increased (Figures 1B,C). The expression levels of CASC2 were reduced after transfection with si-CASC2 (Figures 1D,E). Upon DNA damage, H2A.X is phosphorylated on serine 139, and phosphorylated H2A.X (p-H2A.X, also termed γH2A.X) usually serves as a marker of DNA damage (16, 17). To determine whether CASC2 overexpression induces DNA damage, p-H2A.X was detected using western blot analysis. The levels of p-H2A.X were increased in TE-1 and EC9706 cells 48 after transfection with pcDNA3.1-CASC2 (Figures 1F,G), suggesting that CASC2 overexpression induces DNA damage in ESCC cells.

### Overexpression of LncRNA CASC2 Enhanced Cisplatin-Induced Viability Inhibition in ESCC Cells

**Figure 2:**
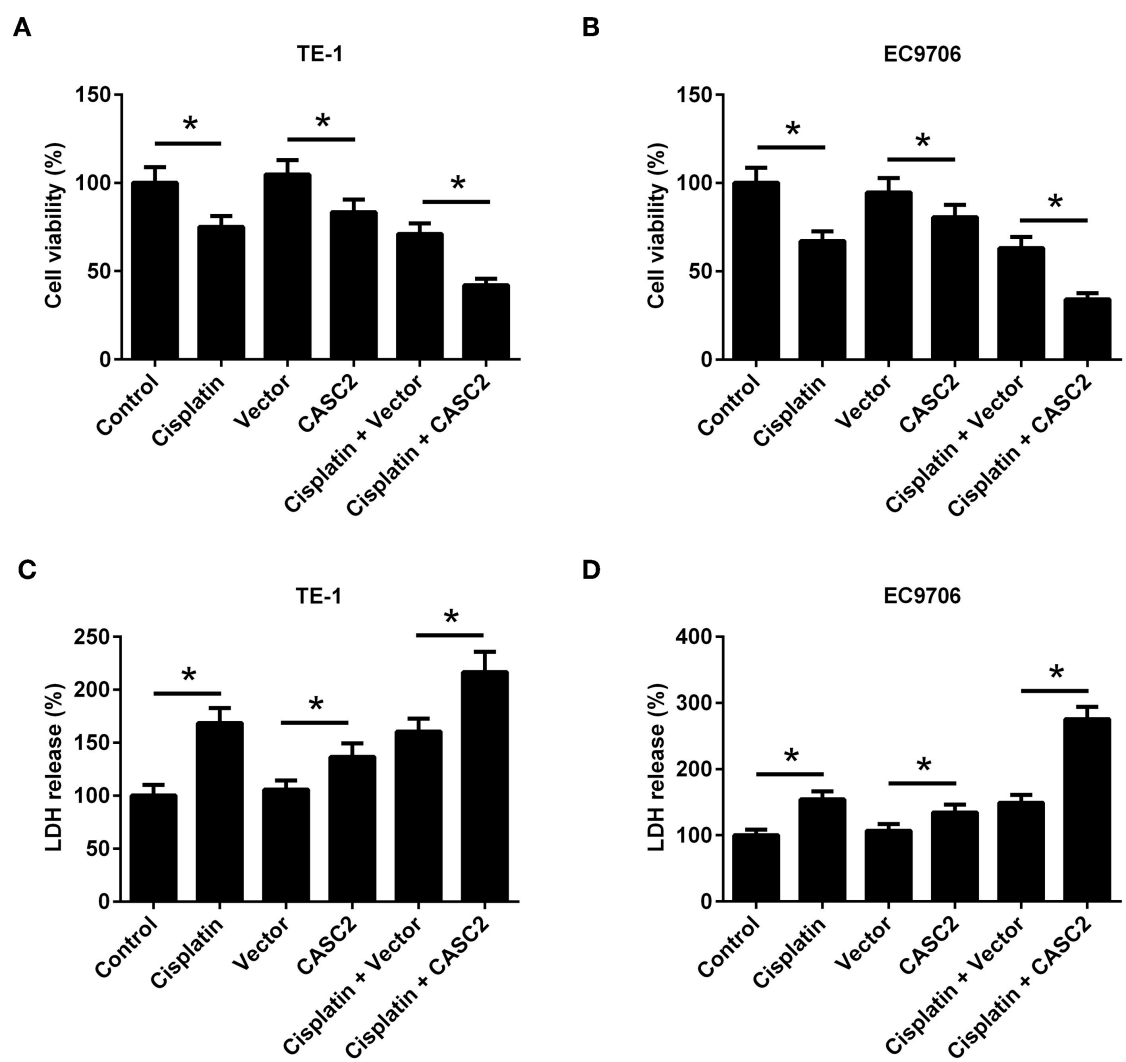
Overexpression of lncRNA CASC2 enhanced cisplatin-induced viability inhibition in ESCC cells. TE-1 and EC9706 cells were transfected with pcDNA3.1-CASC2 (CASC2) or pcDNA3.1 (Vector). Cells were treated with cisplatin (5 μM) for 48 h. **(A,B)** The viability of TE-1 and EC9706 cells. **(C,D)** LDH release of TE-1 and EC9706 cells. ^*^p < 0.05, *n* = 3.

As shown in Figures 2A,B, cisplatin or CASC2 overexpression inhibited cell viability of TE-1 and EC9706 cells. To investigate the role of CASC2 in cisplatin-induced viability inhibition, pcDNA3.1-CASC2 was transfected into TE-1 and EC9706 cells. We found that CASC2 enhanced the inhibitory effect of cisplatin on cell viability (Figures 2A,B). Besides, cisplatin or CASC2 overexpression induced LDH release in TE-1 and EC9706 cells, and CASC2 increased the induction by cisplatin (Figures 2C,D).

### Overexpression of LncRNA CASC2 Enhanced Cisplatin-Induced Apoptosis of ESCC Cells

**Figure 3:**
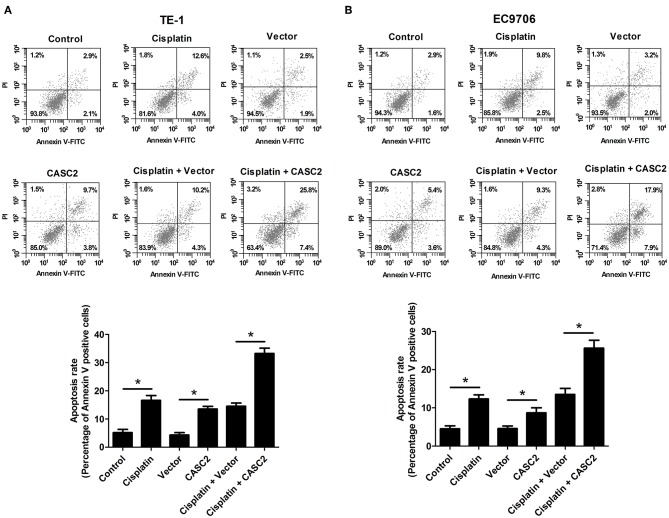
Overexpression of lncRNA CASC2 enhanced cisplatin-induced apoptosis of ESCC cells. TE-1 and EC9706 cells were transfected with pcDNA3.1-CASC2 (CASC2) or pcDNA3.1 (Vector). Cells were treated with cisplatin (5 μM) for 48 h. The apoptosis rate of TE-1 and EC9706 cells was measured by flow cytometry. **(A)** The apoptosis rate of TE-1 cells. **(B)** The apoptosis rate of EC9706 cells. ^*^p < 0.05, *n* = 3.

In order to determine the effect of CASC2 on cell apoptosis, flow cytometry was performed. The results showed that cisplatin or CASC2 overexpression induced cell apoptosis both in TE-1 and EC9706 cells. CASC2 overexpression enhanced cisplatin-induced apoptosis in TE-1 and EC9706 cells, compared to the cells transfected with empty vector (Figures 3A,B). The results indicated that overexpression of CASC2 enhanced cisplatin-induced apoptosis of ESCC cells.

### CASC2 Knockdown Attenuated the Antitumor Activity of Cisplatin

**Figure 4:**
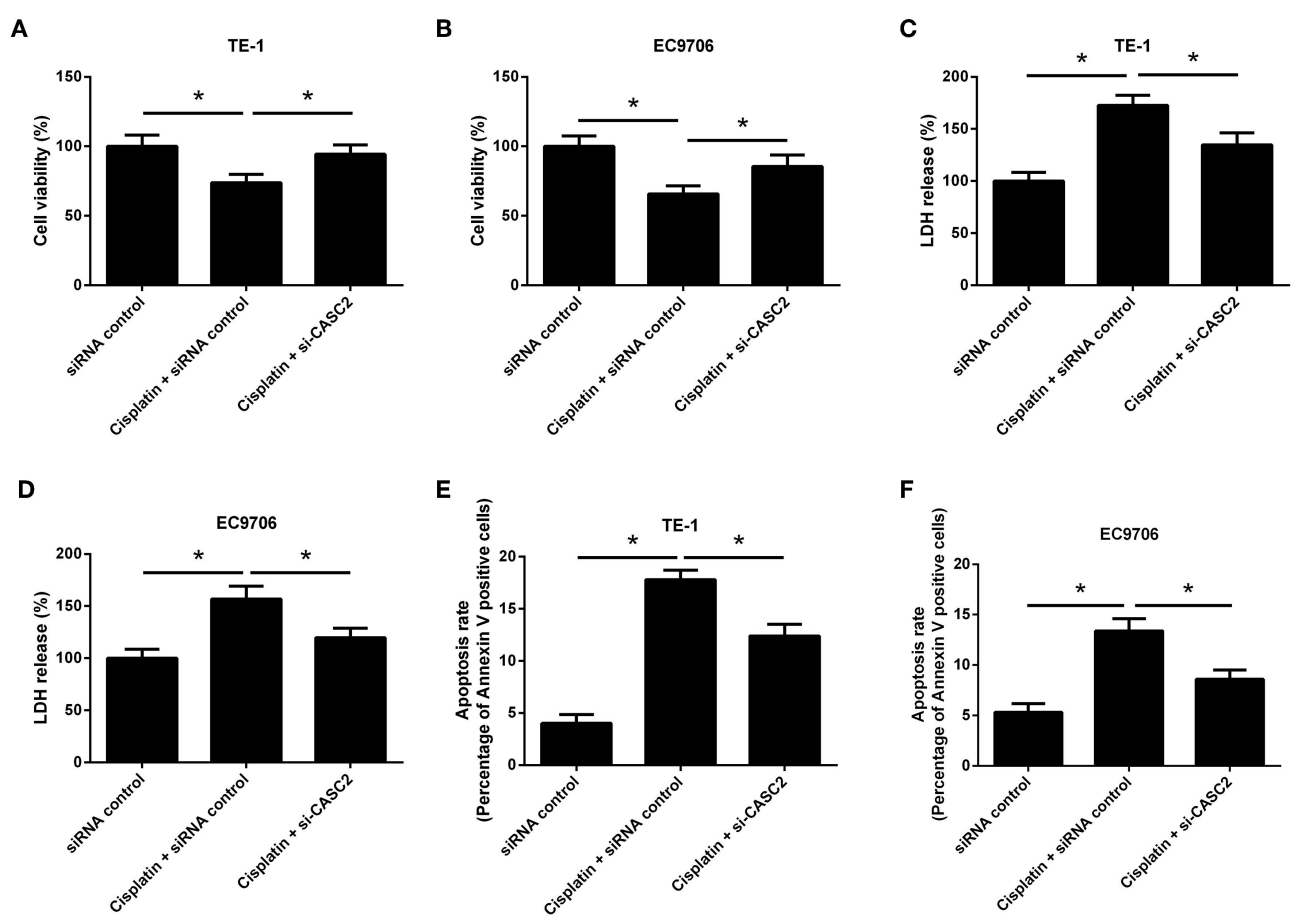
CASC2 knockdown attenuated the antitumor activity of cisplatin. TE-1 and EC9706 cells were transfected with si-CASC2 or siRNA control. Cells were treated with cisplatin (5 μM) for 48 h. **(A,B)** The viability of TE-1 and EC9706 cells was assessed by MTT assay. **(C,D)** LDH release of TE-1 and EC9706 cells was detected using a commercial kit. **(E,F)** The apoptosis rate of TE-1 and EC9706 cells was measured by flow cytometry. ^*^p < 0.05, *n* = 3.

We also determined the effect of CASC2 knockdown on the antitumor activity of cisplatin. As shown in Figures 4A,B, CASC2 knockdown resisted cisplatin-induced viability reduction in TE-1 and EC9706 cells. CASC2 knockdown suppressed cisplatin-induced increase in LDH release in TE-1 and EC9706 cells (Figures 4C,D). Treatment with cisplatin induced apoptosis of TE-1 and EC9706 cells, but this effect was attenuated by CASC2 knockdown (Figures 4E,F). These data suggested that CASC2 knockdown attenuated the antitumor activity of cisplatin.

### MiR-181a Was Up-Regulated and miR-181a Down-Regulation Induced DNA Damage in ESCC Cells

**Figure 5:**
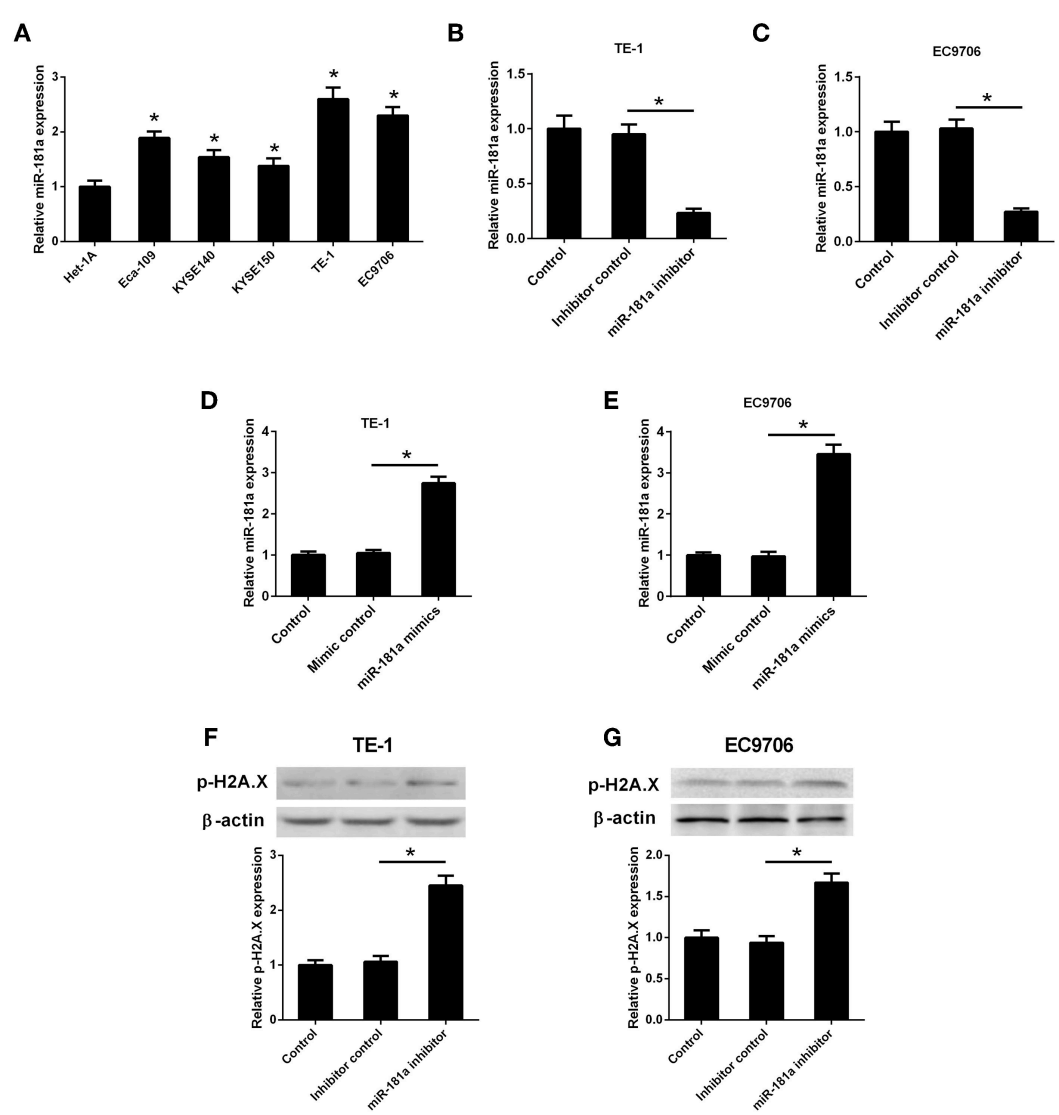
MiR-181a was up-regulated in ESCC cells. **(A)** The expression of miR-181a in normal esophageal epithelial cell line (Het-1A) and human ESCC cell lines (Eca109, KYSE140, KYSE150, TE-1, and EC9706) was detected by qRT-PCR. ^*^p < 0.05 vs. Het-1A cells, *n* = 3. **(B,C)** TE-1 and EC9706 cells were transfected with miR-181a inhibitor or inhibitor control. The expression of miR-181a was detected by qRT-PCR after transfection for 48 h. ^*^p < 0.05, *n* = 3. **(D,E)** TE-1 and EC9706 cells were transfected with miR-181a mimics or mimic control. The expression of miR-181a was detected by qRT-PCR after transfection for 48 h. ^*^p < 0.05, *n* = 3. **(F,G)** The levels p-H2A.X was determined using western blot analysis in TE-1 and EC9706 cells 48 after transfection with miR-181a inhibitor or inhibitor control. ^*^p < 0.05, *n* = 3.

It has been reported that miR-181a expression was up-regulated in ESCC tumor tissues (18). In the present study, we evaluated the expression of miR-181a in ESCC cells. We found that miR-181a expression levels were increased in the ESCC cell lines (Figure 5A). To evaluate the role of miR-181a in ESCC cell lines, the miR-181a inhibitor, inhibitor control, miR-181a mimics, or mimic control was transfected into TE-1 and EC9706 cells. The miR-181a expression levels were significantly decreased in TE-1 and EC9706 cells transfected with miR-181a inhibitor (Figures 5B,C). The miR-181a expression levels were significantly increased in TE-1 and EC9706 cells transfected with miR-181a mimics (Figures 5D,E). To determine whether miR-181a down-regulation induces DNA damage, p-H2A.X levels were determined using western blot analysis. The levels of p-H2A.X were increased in TE-1 and EC9706 cells 48 after transfection with pcDNA3.1-CASC2 (Figures 5F,G), indicating that miR-181a down-regulation induces DNA damage in ESCC cells.

### MiR-181a Down-Regulation Enhanced the Antitumor Activity of Cisplatin

**Figure 6:**
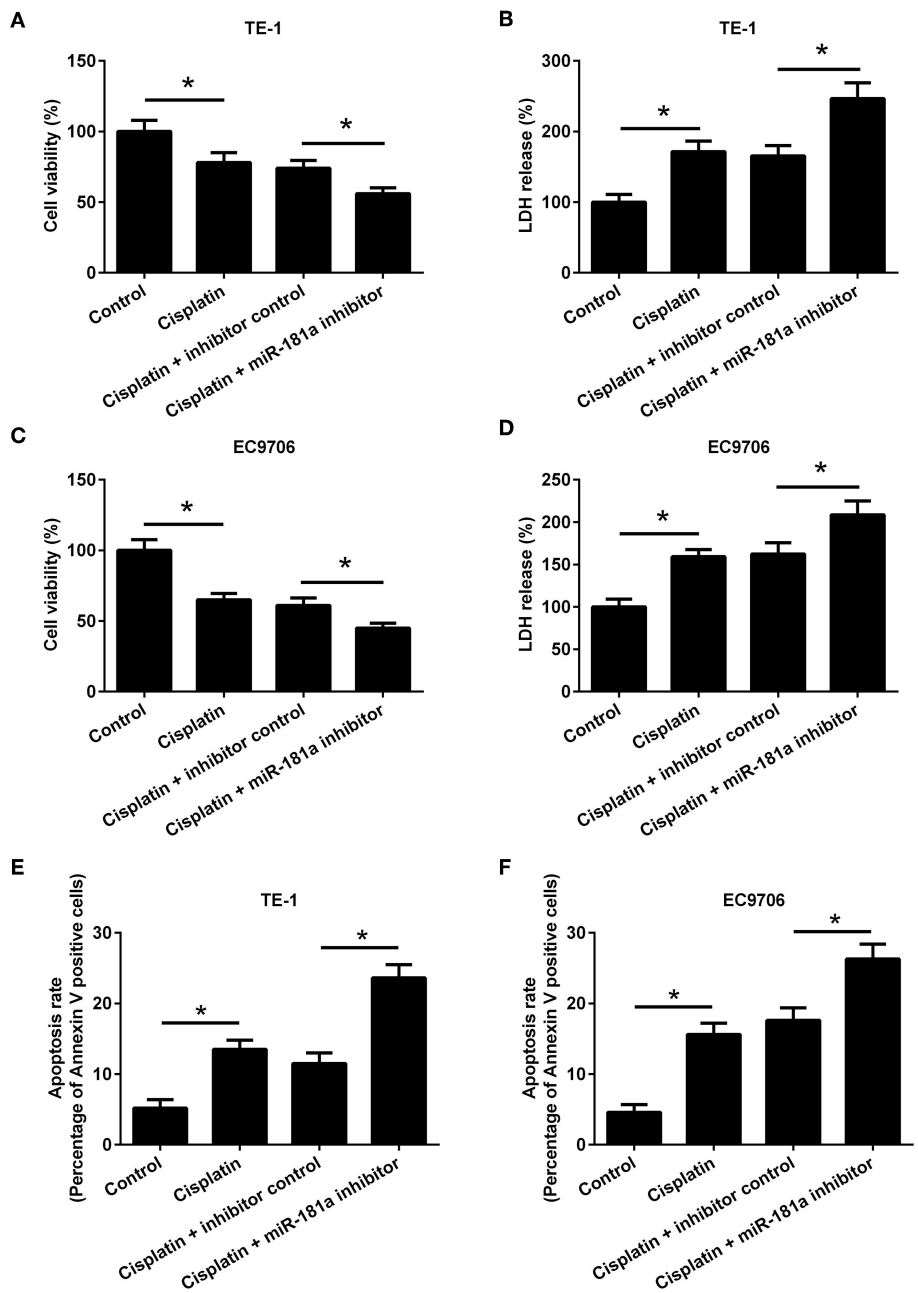
MiR-181a down-regulation enhanced the antitumor activity of cisplatin. TE-1 and EC9706 cells were transfected with miR-181a inhibitor or inhibitor control. Cells were treated with cisplatin (5 μM) for 48 h. **(A,B)** Cell viability and LDH release of TE-1 cells. **(C,D)** Cell viability and LDH release of EC9706 cells. **(E,F)** The apoptosis rate of TE-1 and EC9706 cells was measured by flow cytometry. ^*^p < 0.05, *n* = 3.

We found that cisplatin exhibited antitumor activity by inhibiting cell viability, promoting LDH release, and induced apoptosis of TE-1 and EC9706 cells. To evaluate the effect of miR-181a on the antitumor activity of cisplatin, TE-1, and EC9706 cells were transfected with miR-181a inhibitor or inhibitor control. MiR-181a inhibitor enhanced the inhibitory effect of cisplatin on cell viability (Figures 6A,C). MiR-181a inhibitor also increased cisplatin-induced LDH release (Figures 6B,D). Besides, apoptosis rate was significantly increased in TE-1 and EC9706 cells transfected with miR-181a inhibitor compared to that in cells transfected with inhibitor control in the presence of cisplatin (Figures 6E,F).

### CASC2 Directly Interacted With miR-181a

**Figure 7:**
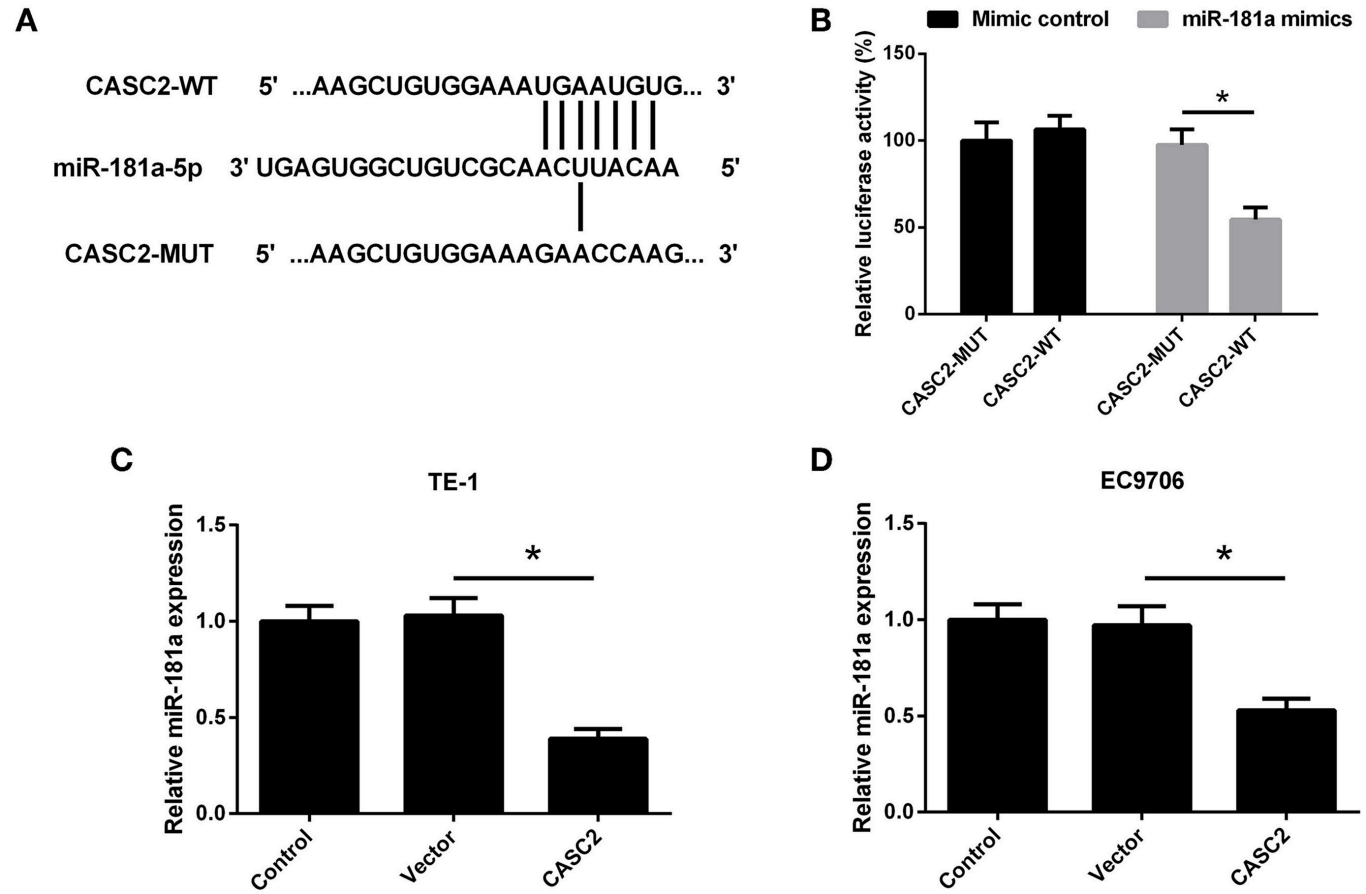
miR-181a was a direct target of CASC2. **(A)** Putative binding sites of CASC2 and miR-181a. **(B)** The relative luciferase activities were detected in TE-1 cells. **(C,D)** TE-1 and EC9706 cells were transfected with pcDNA3.1-CASC2 (CASC2) or pcDNA3.1 (Vector) for 48 h. The expression of miR-181a was determined by qRT-PCR. ^*^p < 0.05, *n* = 3.

A previous study has demonstrated that miR-181a is a direct target of CASC2 and CASC2 negatively regulates miR-181a expression in glioma cells (19). However, it is unknown whether miR-181a is regulated by CASC2 in ESCC cells. The complementary binding sites between lncRNA CASC2 and miR-181a were shown in Figure 7A. As shown in Figure 7B, co-transfection with CASC2-WT and miR-181a mimics decreased the luciferase activity in TE-1 cells. To confirm the effect of CASC2 on miR-181a expression in ESCC cells, TE-1 and EC9706 cells were transfected with pcDNA3.1-CASC2. The miR-181a expression was detected by qRT-PCR. As shown in Figures 7C,D, CASC2 overexpression inhibited the expression of miR-181a in TE-1 and EC9706 cells. These findings suggested that CASC2 directly interacted with miR-181a.

### MiR-181a Resisted the Effects of LncRNA CASC2 on Antitumor Activity of Cisplatin

**Figure 8:**
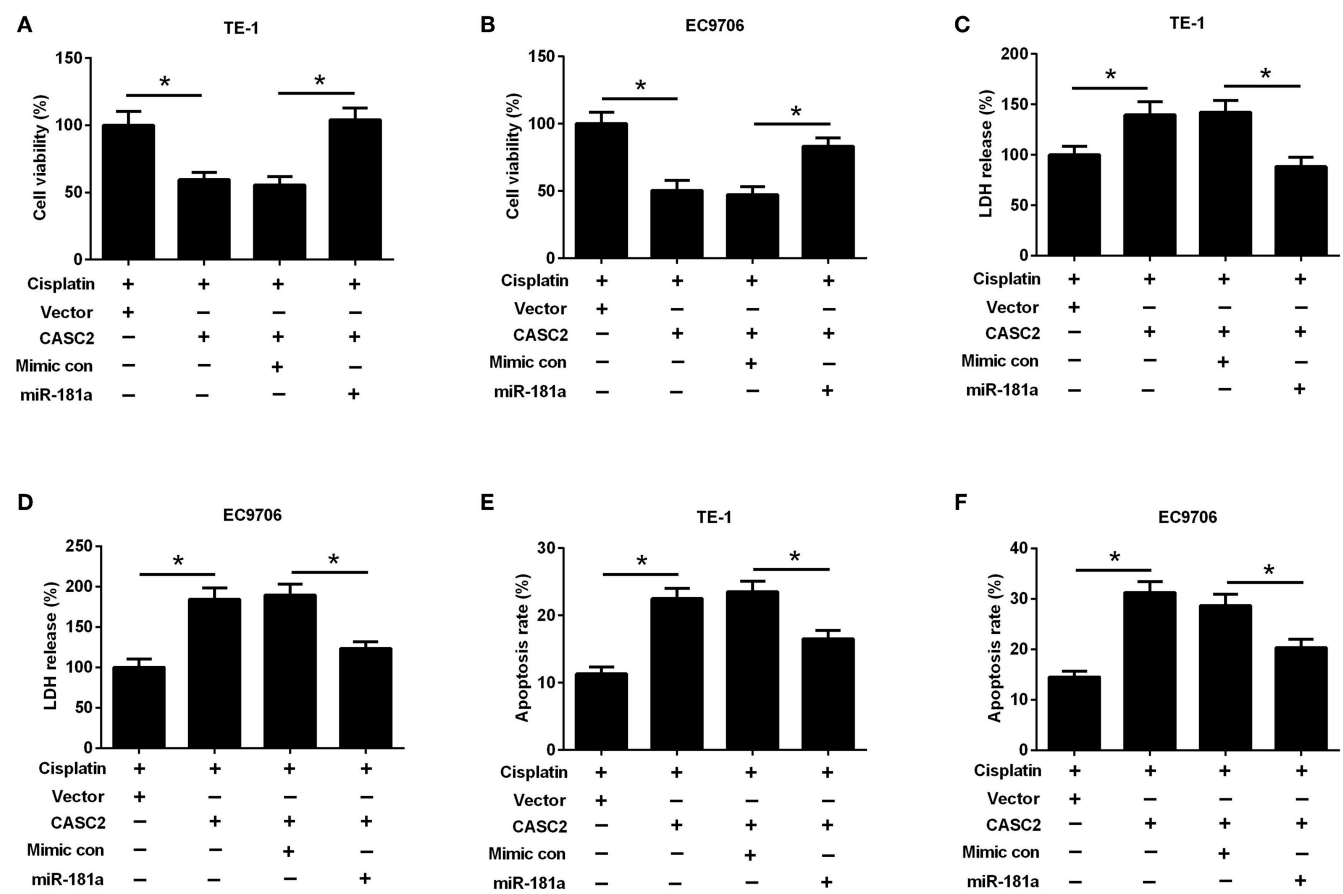
MiR-181a resisted the effects of lncRNA CASC2 on antitumor activity of cisplatin. TE-1 and EC9706 cells were transfected with pcDNA3.1-CASC2 and/or miR-181a mimics, and treated with or without cisplatin (5 μM) for 48 h. Cell viability of TE-1 **(A)** and EC9706 **(B)** cells was detected by MTT assay. LDH release of TE-1 **(C)** and EC9706 **(D)** cells was determined by LDH release assay. The apoptosis rate of TE-1 **(E)** and EC9706 **(F)** cells was measured by flow cytometry. ^*^p < 0.05, *n* = 3.

MiR-181a mimics and pcDNA3.1-CASC2 were co-transfected into the cells to investigate the role of miR-181a in the effect of CASC2. As shown in Figures 8A,B, miR-181a overexpression reversed the effect of CASC2 on cell viability. The LDH release was decreased in cells co-transfected with miR-181a mimics and pcDNA3.1-CASC2, compared with cells co-transfected with mimics control, and pcDNA3.1-CASC2 (Figures 8C,D). The apoptosis rate was reduced in cells co-transfected with miR-181a mimics and pcDNA3.1-CASC2, compared with cells co-transfected with mimics control and pcDNA3.1-CASC2 (Figures 8E,F). All these finding suggested that CASC2 enhanced antitumor activity of cisplatin by inhibiting miR-181a expression.

### LncRNA CASC2 Suppressed the Akt Pathway by Inhibition of miR-181a

**Figure 9:**
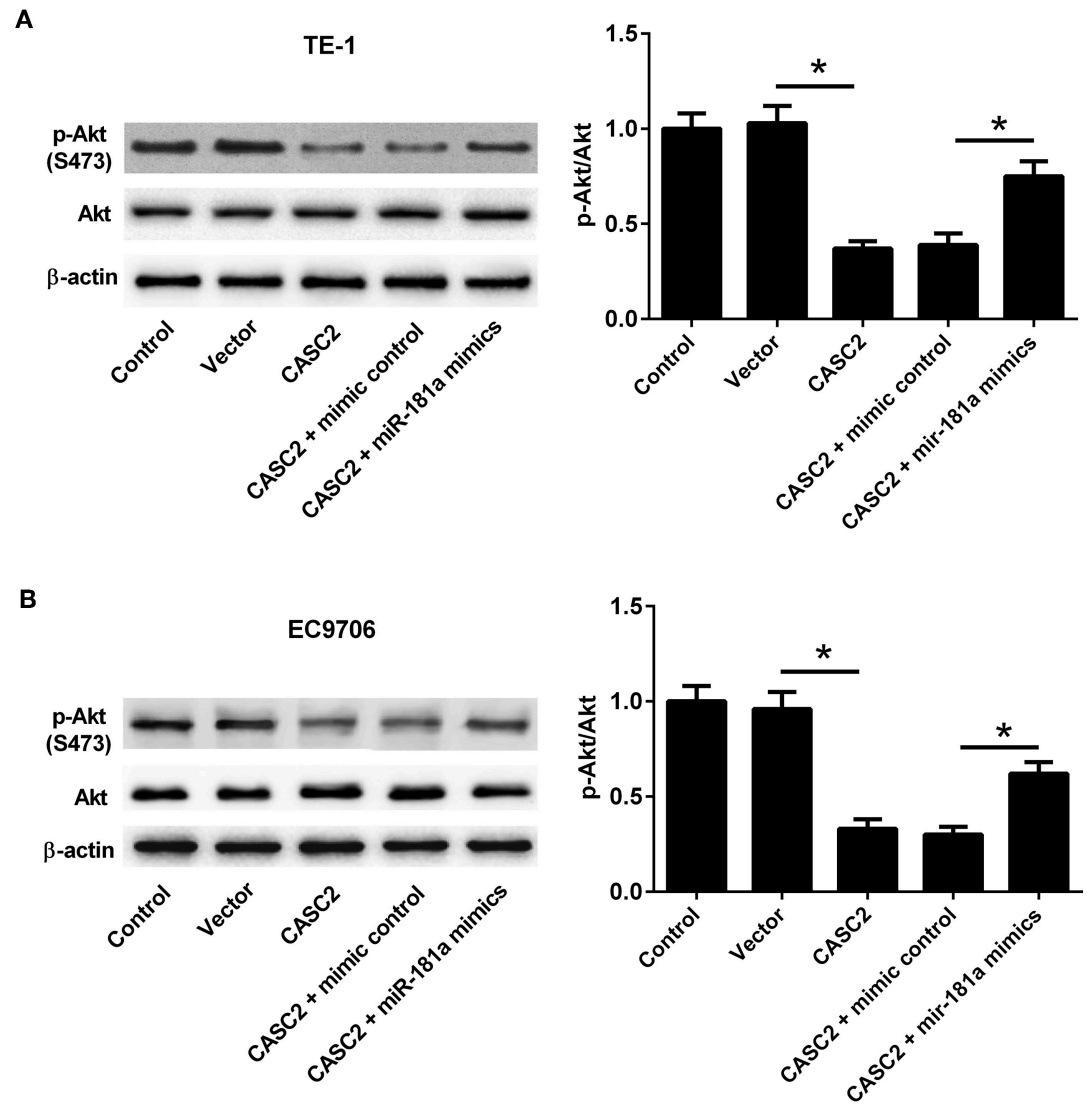
LncRNA CASC2 suppressed the Akt pathway by inhibition of miR-181a. **(A,B)** TE-1 and EC9706 cells were transfected with pcDNA3.1-CASC2 and/or miR-181a mimics for 48 h. The expression levels of p-Akt and Akt were analyzed by western blotting. ^*^p < 0.05, *n* = 3.

Activation of Akt is one of the most common molecular alterations in cancer (20). Akt pathway has been considered as an important therapeutic target for ESCC treatment (21). To investigate whether Akt pathway was involved in the effect of lncRNA CASC2, the expression levels of Akt and p-Akt were measured by western blotting. We found that CASC2 overexpression significantly inhibited the p-Akt expression in TE-1 and EC9706 cells. However, miR-181a overexpression increased the expression level of p-Akt, which was inhibited by CASC2 overexpression (Figures 9A,B). The results suggested that CASC2 suppressed the Akt pathway by inhibiting miR-181a expression.

## Discussion

CASC2, a recently discovered lncRNA, has been demonstrated to play roles in several human cancers (22–24). A previous study reported that the expression of CASC2 was reduced in the hepatocellular carcinoma (HCC) tissues compared with the normal tissues. CASC2 overexpression inhibited growth and induced apoptosis of HCC cells. The expression of CASC2 was negatively related to miR-24-3p expression in the HCC tissues and CASC2 could negatively regulate the expression of miR-24-3p *in vitro* (7). The role of CASC2 in ESCC was reported in a recent published article. The expression of CASC2 was down-regulated both in ECCC tissues and ECCC cell lines. Low expression of CACS2 was associated with advanced TNM stage and lymph node metastases. Moreover, overexpression of CASC2 significantly inhibited proliferation, suppressed invasion, and induced apoptosis of ECCC cells (10).

Chemotherapeutic insensitivity is a major obstacle for treatment of many cancers. It has been demonstrated that CASC2 was low-expressed in the cisplatin-resistant cervical cancer tissues compared to cisplatin-sensitive cancer tissues and overexpression of CACS2 sensitized cisplatin-resistant cervical cancer cells to cisplatin (9). However, the role of CASC2 in regulating cisplatin-treated ESCC cells remains to be identified. In the present study, we evaluated the effect of CASC2 on the cisplatin-treated ESCC cells. We found that CASC2 overexpression enhanced the antitumor effect of cisplatin by inhibiting cell viability, promoting LDH release, and inducing cell apoptosis.

MiR-181a is a member of miR-181 family that is highly conserved in the seed-region sequence, suggesting that miR-181 family may have redundancy in targeting genes (25). Altered expression of miR-181a has been discovered in several types of cancer, such as breast (26), prostate (27), and colon cancer (28). Xiang et al. reported that the expression of miR-181a was significantly increased in ESCC tissues compared with normal adjacent tissues. The miR-181a expression was also significantly up-regulated in patients with lymph node metastasis. MiR-181a up-regulation was associated with advanced TNM stage in ESCC, suggesting that miR-181a might play a crucial role in the development or pathogenesis of ESCC (18). Ba et al. proved that CASC2 suppressed miR-181a expression in osteosarcoma cells (29). In the present study, we also found that CASC2 negatively regulated miR-181a in ESCC cells. Besides, miR-181a inhibitor exhibited the similar effect with CASC2, indicating that CASC2 regulated cisplatin-treated cells through regulating miR-181a.

Akt is a serine/threonine-specific protein kinase that plays an important role in multiple cellular processes, including cell apoptosis, proliferation, and migration (30). Akt and its upstream regulators are deregulated in many types of solid tumors and hematologic malignancies (30). Therefore, the Akt pathway is a key determinant of biologic aggressiveness of various cancers, and it is considered as a major potential target for novel anti-cancer therapies (30, 31). Due to the function of Akt pathway described above, we investigated whether the Akt pathway was involved in the effect of CASC2/miR-181a on cisplatin cytotoxicity. PTEN was reported to be a direct target of miR-18a and miR-21. CASC2 negatively regulated miR-18a and miR-21, thus modulating PTEN/phosphoinositide 3-kinase (PI3K)/Akt pathway and cisplatin-induced viability inhibition in non-small cell lung cancer (32). Overexpression of CASC2 inhibited epithelial ovarian cancer development by inhibiting EIF4A3 expression and suppressing the PI3K/AKT/mammalian target of rapamycin (mTOR) pathway (33). Interestingly, a previous study demonstrated that miR-181a directly targeted PTEN, leading to an increase in phosphorylated AKT in colon cancer cells (34). In the present study, we found that CASC2 suppressed the Akt pathway in ESCC cells by inhibiting miR-181a. The results indicated that CASC2 enhanced the antitumor activity of cisplatin through suppressing the Akt pathway.

## Conclusion

In summary, the role of CASC2 in cisplatin-treated ESCC cells was evaluated. The results suggested that CASC2 was low-expressed in ESCC cell lines. Overexpression of CASC2 enhanced the antitumor effect of cisplatin by inhibiting cell viability, promoting LDH release, and inducing cell apoptosis. CASC2 negatively regulated the expression of miR-181a. MiR-181a inhibitor exhibited the similar effect with CASC2. We also found that CASC2 suppressed the Akt pathway by inhibiting miR-181a. The results indicated that the CASC2/miR-181a/Akt axis might be a potential new target for treatment of ESCC.

## Author Contributions

The research was conceived and designed by DZ and SZ. The experiments were carried out by DZ, CZ, and YYa. The data was analyzed by YYu, YQ, KW, and DL. The manuscript was written by DZ.

### Conflict of Interest Statement

The authors declare that the research was conducted in the absence of any commercial or financial relationships that could be construed as a potential conflict of interest.
